# Prelacteal feeding practice and its associated factors among mothers having children less than 2 years of age in East Africa: a multilevel analysis of the recent demographic and health surveys

**DOI:** 10.1186/s13006-021-00414-z

**Published:** 2021-09-08

**Authors:** Achamyeleh Birhanu Teshale, Misganaw Gebrie Worku, Zemenu Tadesse Tessema, Getayeneh Antehunegn Tesema

**Affiliations:** 1 Department of Epidemiology and Biostatistics, Institute of Public Health, College of Medicine and Health Sciences, University of Gondar, Gondar, Ethiopia; 2 Department of Human Anatomy, University of Gondar, College of Medicine and Health Science, School of Medicine, Gondar, Ethiopia

**Keywords:** Prelacteal feeding, East Africa, Multilevel analysis

## Abstract

**Background:**

Prelacteal feeding is a major public health problem that increases the risk of morbidity and mortality in children. It also result delayed breastfeeding initiation and interferes with exclusive breastfeeding. Although numerous studies have been done on prelacteal feeding in individual East African countries, most of them did not consider community-level factors that could affect the likelihood of prelacteal feeding. This study, thus, aimed to assess the pooled prevalence and associated factors of prelacteal feeding practice in East Africa.

**Methods:**

We used pooled data from the 12 east Africa countries Demographic and Health Surveys (DHS). A total weighted sample of 33,423 women was included in the final analysis. We employed multilevel logistic regression analysis to assess factors associated with prelacteal feeding practice. Finally, the Adjusted odds ratio (AOR) with 95% Confidence (CI) interval was reported and variables with *p* value< 0.05, in the multivariable analysis, were declared to be significant predictors of prelacteal feeding practice.

**Result:**

In this study, the pooled prevalence of prelacteal feeding practice was 11.85% (95%CI: 11.50, 12.20) with great variation between countries, ranging from 3.08% (95%CI: 2.35, 3.81) in Malawi to 39.21% (95%CI: 36.36, 42.06) in Comoros. Both individual and community-level factors were associated with prelacteal feeding practice. Of the individual-level factors, home delivery, multiple birth, cesarean delivery, non-exposure to media, delayed initiation of breastfeeding, and being a small-sized baby were associated with higher odds of prelacteal feeding practice. Among the community-level factors, rural residence and higher community-level of media exposure were associated with lower odds of prelacteal feeding practice.

**Conclusion:**

In this study, the pooled prevalence of prelacteal feeding is high. Both individual and community level variables were associated with prelacteal feeding practice. Therefore, individual and community-level interventions that encourage mothers to deliver in the health facility and promoting timely initiation of breastfeeding are needed to reduce prelacteal feeding practices in east Africa. Moreover, media campaigns regarding this harmful traditional practice could be strengthened.

## Background

Optimal breastfeeding is important for the immediate and long-lasting health of the child by preventing the most childhood killers: pneumonia and diarrhea [1]. Practicing optimal breastfeeding is very important for the prevention of undernutrition and the cognitive development of the newborn [2–4]. Moreover, optimal breastfeeding significantly reduces the risk of developing different infectious diseases and non-infectious inflammatory diseases such as allergy and asthma, as well as obesity and chronic non-communicable diseases such as diabetes mellitus [5, 6].

Initiating breastfeeding within 1 h of birth, exclusively breastfed for the first 6 months of life, and continued breastfeeding up to the age of 2 years are recommended by the World Health Organization and United Nations Children’s Fund [1]. However, in different countries including countries in East Africa, the majority of mothers offer suboptimal breastfeeding practices to their newborns [7–16].

Prelacteal feeding is giving foods or liquids (except recommended medications) to newborns before breastfeeding is established [1]. It is a major public health problem that increases the risk of acquiring respiratory tract infections, diarrhea, and malnutrition [5, 17]. Furthermore, the practice of prelacteal feeding deprives newborns of taking colostrum that is rich in nutrients and immunoglobulins [18, 19]. It has also shown that giving prelacteal foods delays breastfeeding initiation and interferes with exclusive breastfeeding [1, 3, 20, 21].

Despite its great effects on the health of the newborn, prelacteal feeding is widely practiced in many countries in the world with the highest prevalence in the southeast and central Asia, and Latin America [7, 21–23]. In Africa, most of mothers provide prelacteal foods to their newborn, and in sub-Saharan Africa, about 32.2% newborns are exposed to prelacteal foods [15, 24, 25]. Works of the literature revealed that maternal education [22, 26, 27], antenatal care (ANC) utilization [15, 27], home delivery [13, 28], delivery by cesarean section [14, 22, 27], sex of the child [15, 28], and late initiation of breastfeeding [28] are among the factors that are associated with prelacteal feeding practice.

Although numerous studies are done on prelacteal feeding practice in individual east African countries, most of them did not consider the community-level factors that could affect the likelihood of prelacteal feeding. Therefore, we aimed to assess the pooled prevalence and associated factors of prelacteal feeding practice in east Africa. Identifying various factors at both individual and community levels can have a key role in implementing policies and programs aimed at minimizing prelacteal feeding practices.

## Methods

### Data source, data collection, and study population

We used pooled data from the 12 east Africa countries Demographic and Health Surveys (DHS) that were conducted from 2008 to 2019. All these surveys used a stratified two-stage cluster sampling technique. The key demographic and health indicators were collected in each DHS [29]. Five questionnaires: the Household Questionnaire, the Woman’s Questionnaire, Man’s Questionnaire, the Biomarker Questionnaire, and the Health Facility questionnaire were used in each survey to collect the demographic and health indicators. A pre-test was performed before collecting the data and a debriefing session was held with the pre-test field staff [30]. Further information regarding the data collection procedure is found in each countries survey report.

**Fig. 1:**
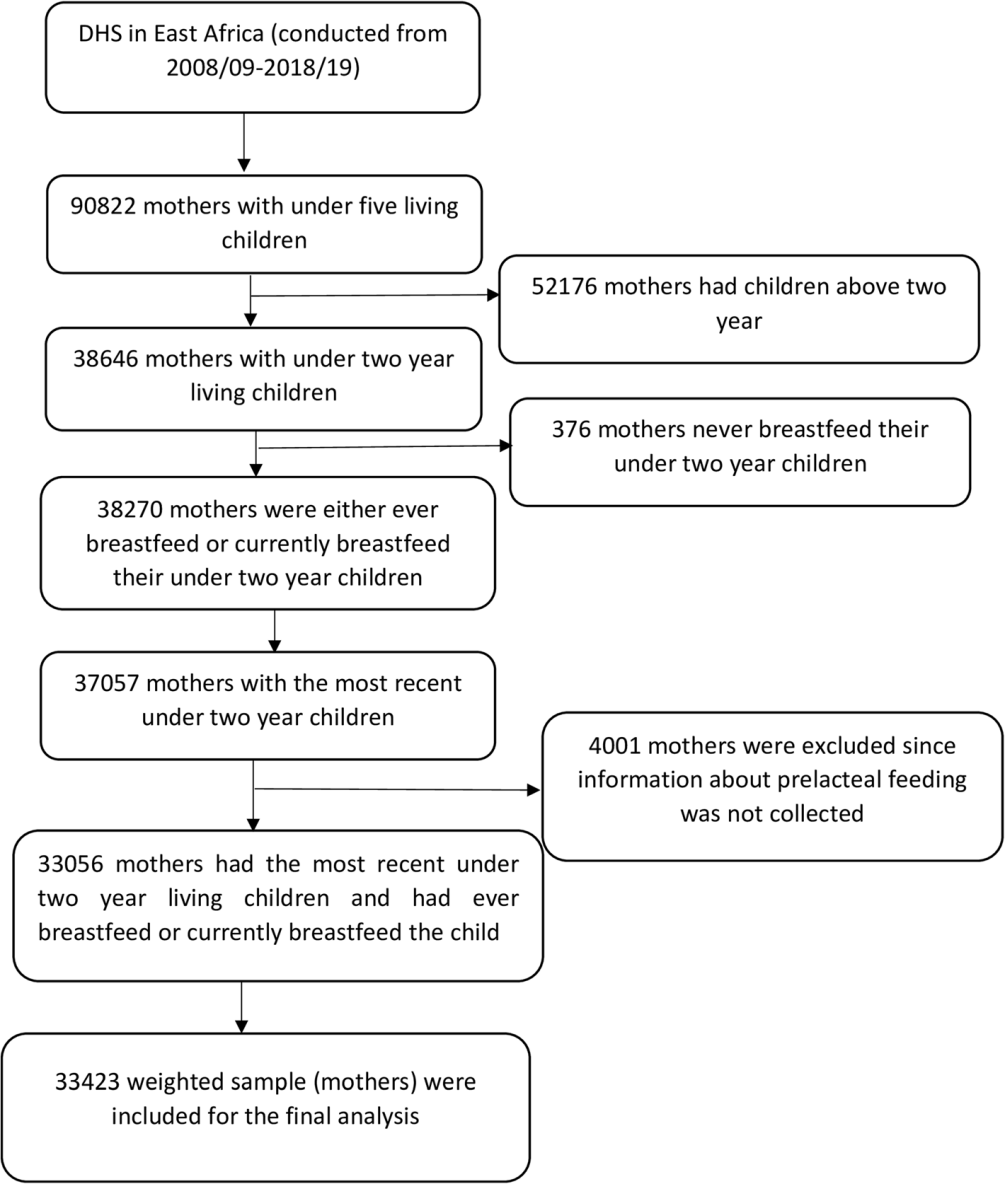
Schematic presentation of how the study sample was selected

For our study, we used a kid’s data set with a total weighted sample of 33,423 women (Fig. 1).

### Variables of the study

#### Dependent variable

The outcome variable was prelacteal feeding practice, which is defined as giving anything other than breast milk for the newborn in the first 3 days after delivery [31].

#### Independent variables

Both individual and community level explanatory variables were incorporated in this study (Table 1).

**Table 1:** Description and categorization of independent variables

Variables	Description/categorization of independent variables
Individual-level variables	
Women age	It was the current age of the mother and grouped into seven categories: 15–19 years, 20–24 years, 25–29 years, 30–34 years, 35–39 years, 40–44 years, and 45–49 years.
Educational level	It was the minimum level of education the mother achieve and it was coded as no formal education, primary education, and secondary and above (combining secondary and higher education categories).
Mother’s occupation	It was the current working status of a woman, which was grouped into non-working and working.
Wealth quantile	In the DHS, it was based on wealth quintiles, with the first (lowest) quintile being the poorest, second (poorer), middle, fourth (rich), and the highest quintile being the richest. For our study, it was re-categorized as poor (includes the lowest and the second wealth quantiles), middle, and rich (includes the fourth and the highest wealth quantiles).
ANC visit	It was defined as the number of ANC visits while a mother was pregnant and it was coded as no ANC visit, 1–2 ANC visits, 3 ANC visits, and 4 and above ANC visits.
Place of delivery	Grouped into delivery at home and health facility.
Delivery by cesarean section	Categorized as yes (if the mother gave birth by CS) and no (otherwise).
Timing of initiation of breastfeeding	It was the time at which the mother gave breast milk to the newborn baby and categorized as early (at/within 1 h) and delayed (after 1 h).
Perception of distance from the health facility	It was based on whether the mother perceives distance from the health facility as a big problem or not a big problem
media exposure	Created by combining whether a respondent reads a newspaper, listens to the radio, and watches television and coded as yes (if a woman had been exposed to at least one of these media) and no (otherwise).
Parity	Categorized as Primiparous (having parity of one), multiparous (having parity of 2–4), and grand multiparous (having parity of 5 and above).
Multiple birth	It was coded as no (if women gave a single birth) and yes (otherwise)
Sex of the child	The sex of the child was categorized into female and male.
Size of the child at birth	It was based on the mother’s perception and categorized as very small, smaller than average, average, larger than average, and very large in the DHS. This variable was re-categorized into small (very small, smaller than average), average (average), and large (larger than average and very large) for our study.
Community-level variables	
Residence	The place of residence for the mother and coded as rural and urban
Community-level women education	It was the proportion of women with a minimum of primary level of education derived from data on mothers or respondent’s level of education. Then, it was categorized using national median value to values: low (if the mother was from communities in which ≤50% of women had at least primary education) and high (if the mother was from communities in which > 50% of women had at least primary education) community educational level.
Community poverty level	It was the proportion of women in the poorest and poorer quintiles derived from data on the household wealth index. Then, it was categorized, based on national median value, into: low (if the mother was from communities in which ≤50% of women had poor socioeconomic status) and high (if the mother was from communities in which > 50% of women had poor socioeconomic status) community poverty level.
Community-level of ANC utilization	It was the proportion of women with at least one ANC visit and categorized using national level quartiles to: low (if the mother was from communities in which ≤25% of women utilizing ANC), middle (if the mother was from communities in which 25–75% of women utilizing ANC), and high (if the mother was from communities in which ≥75% of women utilizing ANC) community-level ANC utilization.
Community-level media exposure	The proportions of mothers who were exposed to media within a specific cluster. It was categorized in the same fashion as the community level of women’s education into low and high community-level of media exposure.

Note: ANC = Antenatal Care, CS=Cesarean Section, DHS=Demographic and Health Surveys

##### Individual-level variables

Women age, educational level, maternal occupation, wealth quantile, ANC visit, place of delivery, delivery by cesarean section, the timing of initiation of breastfeeding, perception of distance from the health facility, media exposure, parity, sex of the child, birth order, and size of the child at birth.

##### Community-level variables

In this study, place of residence was a non-aggregate community-level variable while community level of women education, community level of media exposure, community level of ANC utilization, and community poverty level were constructed through the aggregation of individual-level factors to conceptualize their neighborhood effect on prelacteal feeding practice.

### Data management and statistical analysis

The data were appended, recoded, and analyzed using Stata 14 software. The sample was weighted using the primary sampling unit variable, stratification variable, and the weight variable, to restore its representativeness and to get a better estimate throughout the analysis [32]. Both the weighted and unweighted results were presented and compared. The proportion of prelacteal feeding practice per each independent variable and the absolute risk difference was calculated. The pooled data have a hierarchical structure with individuals nested within clusters. Therefore, we employed a multilevel logistic regression analysis. To conduct the multilevel logistic regression analysis, four models were fitted. These are: the null model (a model containing only the outcome variable), model 1 (a model with the outcome variable and individual-level variables), model 2 (a model with the outcome variable and the community variables only), and model 3 (a model with the outcome variable and both the individual and community level variables).

The random effect analysis that is a measure of variation of prelacteal-feeding practice across communities or clusters, were assessed using intra-class correlation coefficient (ICC), median odds ratio (MOR), and a proportional change in variance (PCV) [33–35]. Since the models fitted are nested models, deviance was used for model comparison and model fitness.

Both bivariable and multivariable multilevel analyses were done and variables with *p*-value < 0.20 in the bivariable analysis were eligible for multivariable analysis. Finally, the Adjusted Odds Ratio (AOR) with 95% Confidence Interval (CI) was reported and variables with *p* value< 0.05, in the multivariable analysis, were declared to be significant predictors of prelacteal feeding practice. Variance inflation factor (VIF) was used to test Multicollinearity and there was no Multicollinearity between independent variables.

## Results

### Socio-demographic characteristics of the study population

Among 38,270 mothers, with under 2 year living children, who ever breastfeed or are breastfeed their child, 33,423 mothers (weighted) were included for the final analysis (Fig. 1). The majority of the study participants were from Mozambique, Kenya, Tanzania, Zambia, and Ethiopia. Regarding place of residence, more than three fourth (77.23%) of respondents were rural dwellers. The median age of mothers was 27 (IQR ± 10) years. About half (50.36%) of the respondents had a primary level of education and 45.83% of respondents were from households with poor socioeconomic status. Regarding the timing of breastfeeding initiation, the majority (80.67%) of respondents initiate breast milk within 1 h. More than half (51.7%) and three-fourth (78.86%) of respondents had four and above ANC visits and gave their last birth at the health facility, respectively (Table 2).

**Table 2:** Sociodemographic characteristics of respondents and their children

Variables	Unweighted frequency (*N* = 33,056)	Percentage (%)	Weighted frequency (*N* = 33,423)	Percentage (%)
Country				
Burundi	2504	7.58	2579	7.72
Ethiopia	3821	11.56	4050	12.12
Kenya	3659	11.07	3386	10.13
Comoros	1088	3.29	1129	3.38
Madagascar	2326	7.04	2374	7.10
Malawi	2141	6.48	2131	6.37
Mozambique	4247	12.83	4549	13.61
Rwanda	1481	4.48	1507	4.51
Tanzania	4002	12.11	3956	11.84
Uganda	1847	5.59	1807	5.41
Zambia	3757	11.37	3691	11.04
Zimbabwe	2183	6.60	2264	6.77
Maternal age (years)				
15–19	3657	11.06	3734	11.17
20–24	8864	26.82	8824	26.40
25–29	8371	25.32	8522	25.50
30–34	6218	18.81	6299	18.85
35–39	4037	12.21	4104	12.28
40–44	1595	4.83	1610	4.82
45–49	314	0.95	330	0.99
Educational level				
No education	8456	25.58	8467	25.33
Primary	16,222	49.07	16,831	50.36
Sec & above	8378	25.34	8125	24.31
Maternal occupation				
Working	20,829	63.01	21,449	64.17
Not working	12,227	36.99	11,974	35.83
Wealth index				
Poor	15,441	46.71	15,316	45.83
Middle	6047	18.29	6536	19.56
Rich	11,568	35.00	11,571	34.62
Sex of child				
Male	16,522	49.98	16,743	50.09
Female	16,534	50.02	16,680	49.91
Multiple birth				
No	32,578	98.55	32,963	98.62
Yes	478	1.45	460	1.38
Breastfeeding initiation				
Within 1 h	26,642	80.60	26,963	80.67
After 1 h	6414	19.40	6460	19.33
Media exposure				
No	12,167	36.81	12,071	36.12
Yes	20,889	63.19	21,352	63.88
Parity				
Primiparous	6876	20.18	7021	21.01
Multiparous	16,693	50.50	16,812	50.30
Grand multiparous	9487	28.70	9590	28.69
ANC visits				
No	2449	7.41	2678	8.01
One & two	4284	12.96	4378	13.10
Three	8976	27.15	9074	27.15
Four & above	17,347	52.48	17,293	51.74
Place of delivery				
Home	9056	27.40	9072	27.14
Health facility	24,000	72.60	24,351	72.86
Cesarean delivery				
Yes	1966	5.95	1951	5.84
No	31,090	94.05	31,472	94.16
Size of the child at birth				
Small	5569	16.85	5522	16.52
Large	9837	29.76	10,217	30.57
Average	17,650	53.39	17,684	52.91
Distance from the health facility				
Big problem	13,998	42.35	14,769	44.19
Not a big problem	19,058	57.65	18,654	55.81
Residence				
Urban	8318	25.16	7610	22.77
Rural	24,738	74.84	25,813	77.23
Community-level women education				
Low	16,565	50.51	17,369	51.97
High	16,491	49.89	16,054	48.03
Community poverty leve				
Low	16,272	49.23	17,208	51.48
High	16,784	50.77	16,215	48.52
Community-level of ANC utilization				
High	7392	22.36	7044	21.08
Low	11,312	34.22	12,258	36.67
Middle	14,352	43.42	14,121	42.25
Community-level media exposure				
Low	16,255	49.17	16,047	48.01
High	16,801	50.83	17,376	51.99

Note: *ANC* Antenatal Care, *sec* Secondary

### Proportion of prelacteal feeding practice by socio-demographic characteristics and the absolute risk difference

Table 3 revealed the weighted and unweighted proportion of prelacteal feeding by each independent variable and their absolute risk difference. The weighted percentage of prelacteal feeding among mothers who gave a multiple birth was 20.23% while in those who gave single birth was 11.73% with an absolute risk difference of 8.56%. The proportion of prelacteal feeding among mothers who initiated breast milk within an hour and after an hour was 7.99 and 27.93%, respectively, with an absolute risk difference of 19.94%. The absolute risk difference of prelacteal feeding among those mothers who gave birth at home and at the health facility was 8.46%. Regarding country, the highest absolute risk difference (36.13%) was found between Comoros and Malawi (Table 3).

**Table 3:** Proportion of prelacteal feeding by each sociodemographic characteristic and the absolute risk difference

Variables	PF (unweighted)		PF (weighted)		Unweighted Proportion of PF (%)	ARD (%)	Weighted Proportion of PF (%)	ARD (%)
	No	Yes	No	Yes				
Maternal age (years)								
15–19	3152	505	3236	498	13.81	Ref.	13.33	Ref.
20–24	7720	1144	7779	1045	12.91	0.90	11.84	1.47
25–29	7362	1009	7571	951	12.05	1.76	11.15	2.18
30–34	5408	810	5576	723	13.03	0.78	11.48	1.85
35–39	3511	526	3610	494	13.03	0.78	12.48	0.85
40–44	1390	205	1402	208	12.85	0.96	12.04	1.29
45–49	271	43	289	41	13.69	0.12	12.91	0.42
Educational level								
No education	8392	1360	7400	1067	16.08	Ref.	12.60	Ref.
Primary	14,407	1815	14,969	1862	11.19	4.89	11.06	1.54
Sec & above	7311	1007	7094	1030	12.74	3.34	12.68	−0.80
Mother’s occupation								
Working	18,079	2750	18,695	2754	12.20	Ref.	12.84	Ref.
Not working	10,735	1492	10,768	1206	13.20	1.00	10.07	2.77
Wealth index								
Poor	13,295	2146	13,436	1880	13.90	Ref.	12.27	Ref.
Middle	5357	690	5798	738	11.41	2.49	11.29	0.98
Rich	10,162	1406	10,230	1341	12.15	1.75	11.59	0.68
Sex of child								
Male	14,348	2174	14,709	2034	13.16	Ref.	12.14	Ref.
Female	14,466	2068	14,754	1926	12.51	0.65	11.55	0.59
Multiple birth								
No	28,433	4145	29,097	3866	12.72	Ref.	11.73	Ref.
Yes	381	97	367	93	20.29	−7.57	20.29	−8.56
Breastfeeding initiation								
Within 1 h	24,319	2323	24,808	2155	8.71	Ref.	7.99	Ref.
After 1 h	4495	1919	4656	1804	29.92	−21.21	27.93	−19.94
Media exposure								
No	10,606	1561	10,772	1299	12.83	Ref.	10.76	Ref.
Yes	18,208	2681	18,691	2661	12.83	0.00	12.46	−1.70
Parity								
Primiparous	5887	989	6070	951	14.38	Ref.	13.55	Ref.
Multiparous Grand	14,712	1972	14,977	1835	11.81	2.57	10.91	2.64
multiparous	8206	1281	8417	1173	13.50	0.88	12.24	1.31
ANC visits								
No	1890	559	2258	420	22.83	Ref.	15.69	Ref.
One & two	3631	653	3793	585	15.24	7.59	13.35	2.34
Three	7956	1020	8074	1000	11.36	11.47	11.02	4.67
Four & above	15,337	2010	15,338	1955	11.59	11.24	11.30	4.39
Place of delivery								
Home	7173	1883	7438	1634	20.79	Ref.	18.01	Ref.
Health facility	21,641	2359	22,026	2325	9.83	10.96	9.55	8.46
Cesarean delivery								
Yes	1531	435	1527	424	22.13	−9.88	21.72	−10.49
No	27,283	3807	27,937	3535	12.25	Ref.	11.23	Ref.
Size of the child at birth								
Small	4637	932	4732	790	16.74	−4.22	14.32	−2.75
Average	1557	2078	15,697	1987	11.77	0.75	11.23	0.34
Large	8605	1232	9035	1182	12.52	Ref.	11.57	Ref.
Distance from the health facility								
Big problem	12,146	1852	13,005	1764	13.23	Ref.	11.95	Ref.
Not a big problem	16,668	2390	16,459	2195	12.54	0.69	11.77	0.18
Residence								
Urban	7207	1111	6613	997	13.36	Ref.	13.10	Ref.
Rural	21,607	3131	22,851	2962	12.66	0.70	11.48	1.62
Community-level women education								
Low	14,506	2059	15,396	1973	12.43	Ref.	11.36	Ref.
High	14,308	2183	14,068	1986	13.24	−0.81	12.37	−1.01
Community poverty level								
Low	14,100	2172	15,096	2112	13.35	Ref.	12.27	Ref.
High	14,714	2070	14,368	1847	12.33	1.02	11.39	0.88
Community-level of ANC utilization								
High	6430	962	6159	885	13.01	Ref.	12.56	Ref.
Low	10,037	1275	10,980	1278	11.27	1.74	10.43	2.13
Middle	12,347	2005	12,325	1796	13.97	−0.96	12.72	− 0.16
Community-level media exposure								
Low	14,287	1968	14,342	1705	12.11	1.41	10.63	2.34
High	14,527	2274	15,122	2254	13.53	Ref.	12.97	Ref.
Country								
Burundi	2349	155	2440	139	6.19	32.30	5.39	33.71
Ethiopia	3199	622	3728	322	16.28	22.21	7.95	31.21
Kenya	3134	525	2867	519	14.35	24.14	15.31	23.90
Comoros	666	422	686	443	38.49	Ref.	39.21	Ref.
Madagascar	1672	654	1757	617	28.12	10.37	26.00	13.21
Malawi	2078	63	2065	66	2.94	35.35	3.08	36.13
Mozambique	4009	238	4288	261	5.60	32.89	5.75	33.46
Rwanda	1417	64	1441	66	4.32	34.17	4.34	34.87
’Tanzania	3404	598	3411	545	14.94	23.55	13.78	25.43
Uganda	1406	441	1355	452	23.88	14.61	25.01	14.20
Zambia	3544	213	3444	247	5.67	32.82	7.70	31.51
Zimbabwe	1936	247	1981	283	11.31	27.18	12.49	26.72

Note: *PF* Prelacteal Feeding, *ARD* Attributable Risk Difference, *Ref*. Reference

### Prevalence of prelacteal feeding practice in East Africa

**Fig. 2:**
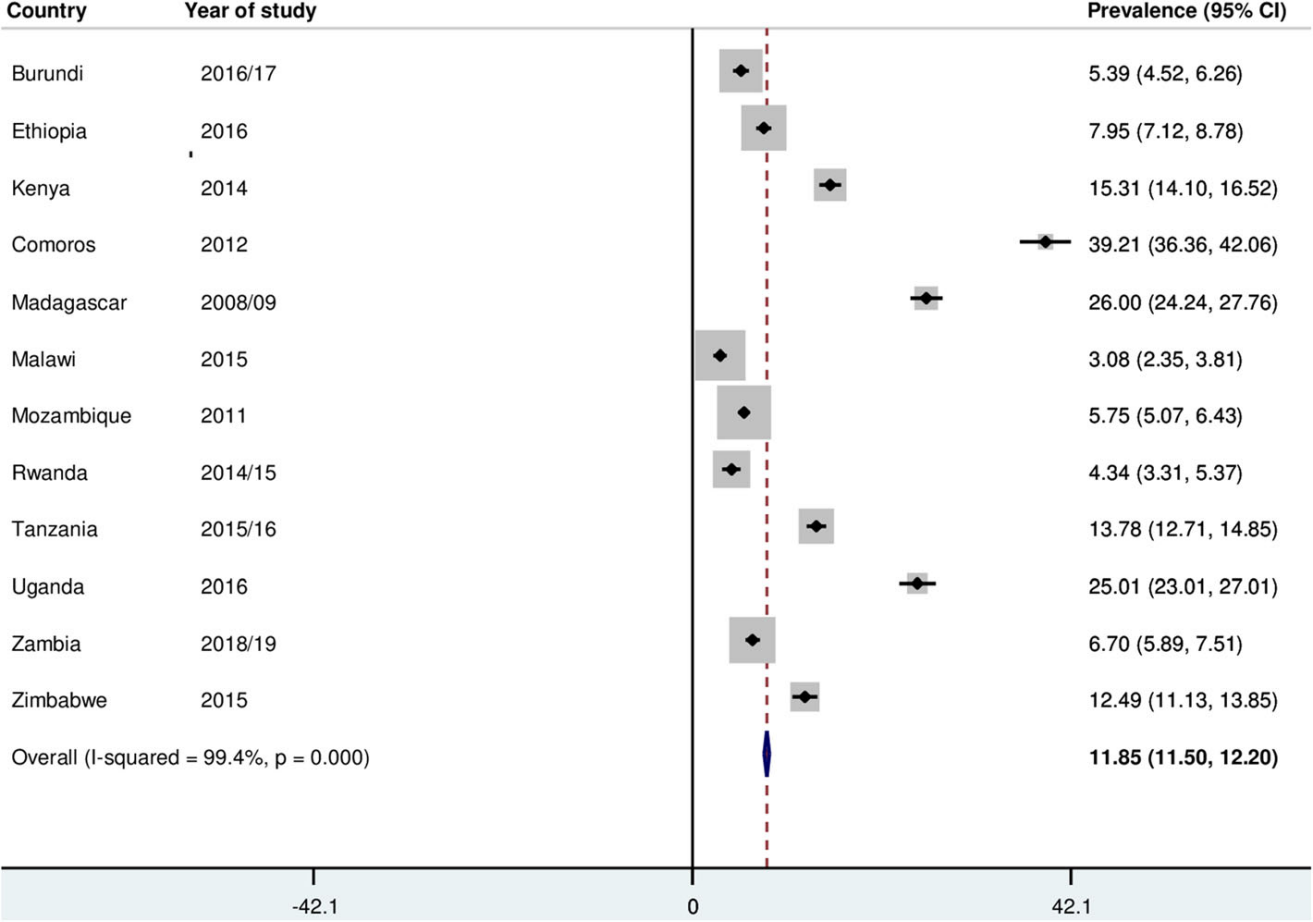
The pooled prevalence of prelacteal feeding practice in East Africa (Weighted)

**Fig. 3:**
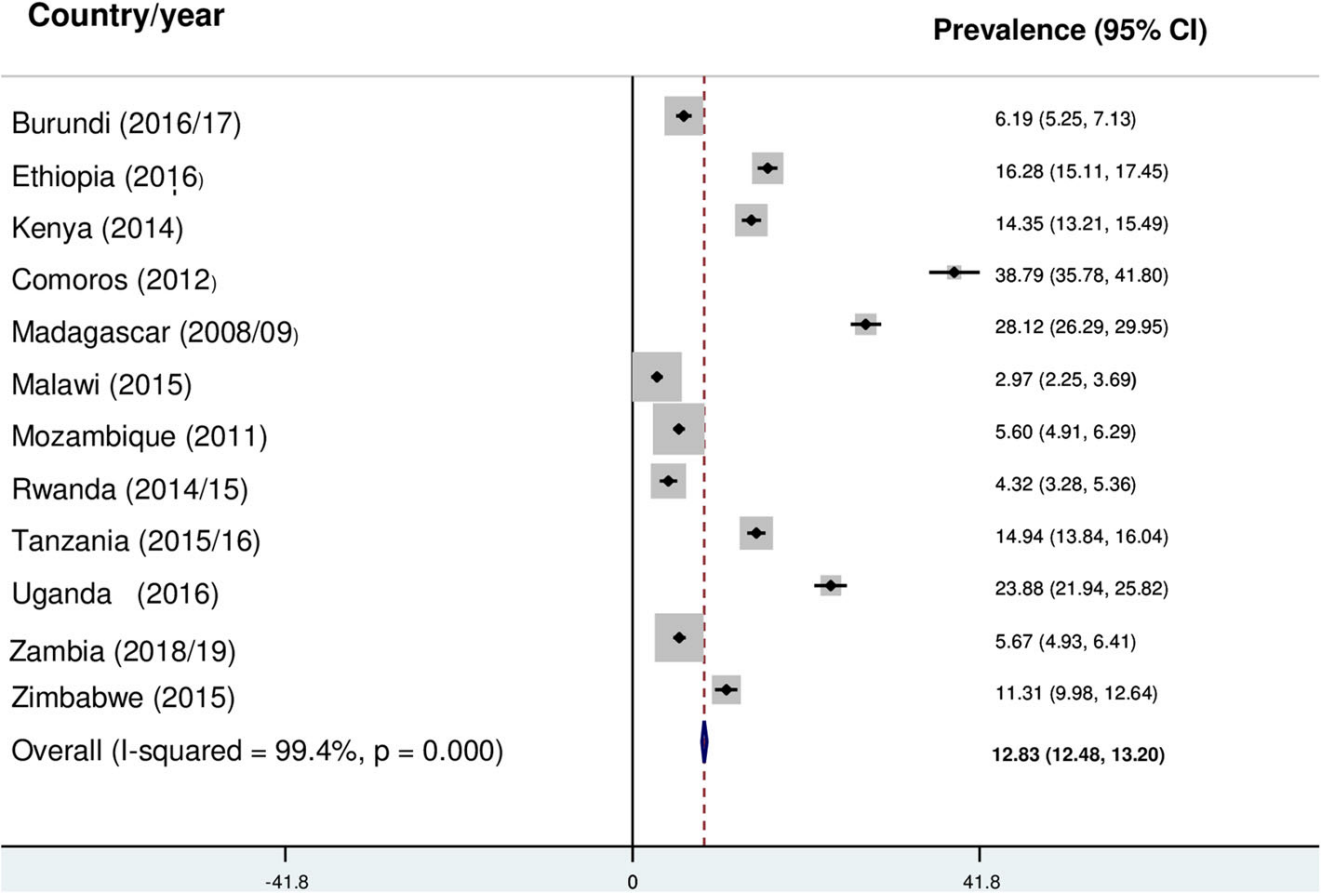
The pooled prevalence of prelacteal feeding practice in East Africa (Unweighted)

The prevalence of prelacteal feeding practice based on the weighted data was 11.85% (95% CI: 11.50, 12.20) with great variation between countries (Fig. 2). However, using the unweighted data, the pooled prevalence was 12.83% (95%CI: 12.48, 13.20) (Fig. 3).

### Factors associated with prelacteal feeding practice in East Africa

#### Fixed effect analysis

Table 4 revealed a multilevel analysis for the final model, both for the weighted and unweighted data. We considered the weighted data to assess factors associated with prelacteal feeding practice in East Africa since it gives an appropriate parameter estimate to draw a valid conclusion. Therefore, the interpretations here are based on the weighted data. In the multivariable multilevel analysis; multiple birth, the timing of breastfeeding initiation, media exposure, place of delivery, delivery by cesarean section, size of the child at birth, residence, and community level of media exposure were significantly associated with prelacteal feeding practice (*p* < 0.05). Mothers who gave a multiple birth had 1.69 [AOR =1.69; 95% CI: 1.22, 1.34] times higher odds of prelacteal feeding practice compared to their counterparts. The odds of practicing prelacteal feeding practice was 3.83 [AOR =3.48; 95% CI: 3.48, 4.23] times higher among mothers who initiated breastfeeding after 1 h compared to their counterparts. Regarding media exposure, mothers who had not been exposed to at least one media had 1.21 [AOR =1.21; 95% CI: 1.07, 1.35] times higher odds of prelacteal feeding practice as compared to those who had exposed to at least one media. Mothers who gave birth in the health facility had 56% [AOR =0.44; 95% CI: 0.39, 0.49] lower odds of prelacteal feeding practice as compared to those who delivered at home. Delivery by cesarean section was also associated with prelacteal feeding in which the odds of prelacteal feeding practice was 1.63 [AOR =1.63; 95% CI: 1.38, 1.93] times higher among mothers who delivered by cesarean section as compared to those who gave vaginal birth. The odds of prelacteal feeding practice was 1.15 [AOR =1.15; 95% CI: 1.01, 1.32] times higher among mothers who gave a small-sized baby as compared to those mothers who gave a large-sized baby. Mothers from the rural area had 22% [AOR =0.78; 95% CI: 0.67, 0.91] lower odds of prelacteal feeding practice as compared to those from urban areas. Regarding community-level of media exposure, mothers from communities with a lower level of media exposure had 1.22 [AOR =1.22; 95% CI: 1.09, 1.36] times higher odds of prelacteal feeding practice as compared to their counterparts (Table 4).

**Table 4:** Factors associated with prelacteal feeding practice in East Africa

Variables	Multilevel Logistic regression			
	Based on unweighted data		Based on weighted data	
	COR (95% CI)	AOR (95% CI)	COR (95% CI)	AOR (95% CI)
Maternal age (years)				
15–19	1.00	1.00	1.00	1.00
20–24	0.91 (0.81, 1.02)	0.92 (0.81, 1.05)	0.87 (0.75, 1.01)	0.90 (0.76, 1.06)
25–29	0.85 (0.76, 0.95)	0.85 (0.74, 0.97) *	0.82 (0.70, 0.95)	0.85 (0.71, 1.02)
30–34	0.93 (0.82, 1.05)	0.92 (0.79, 1.08)	0.84 (0.73, 0.98)	0.87 (0.72, 1.05)
35–39	0.93 (0.82, 1.07)	0.90 (0.76, 1.07)	0.90 (0.75, 1.08)	0.92 (0.73, 1.16)
40–44	0.92 (0.77, 1.10)	0.90 (0.73, 1.12)	0.97 (0.78, 1.21)	0.99 (0.76, 1.29)
45–49	0.98 (0.70, 1.38)	0.92 (0.64, 1.34)	0.94 (0.61, 1.47)	0.93 (0.59, 1.48)
Educational level				
No education	1.00	1.00	1.00	1.00
Primary	0.66 (0.51, 0.72)	0.76 (0.70, 0.83) ***	0.85 (0.76, 0.95)	0.89 (0.78, 1.01)
Sec & above	0.76 (0.69, 0.83)	0.88 (0.79, 0.99)*	0.99 (0.86, 1.13)	1.03 (0.88, 1.20)
Wealth index				
Poor	1.00	1.00	1.00	1.00
Middle	0.80 (0.73, 0.88)	0.93 (0.84, 1.03)	0.91 (0.81, 1.02)	0.97 (0.86, 1.60)
Rich	0.85 (0.79, 0.92)	0.91 (0.82, 1.01)	0.93 (0.84, 1.04)	0.87 (0.76, 1.01)
Sex of child				
Male	1.00	1.00	1.00	1.00
Female	0.94 (0.88, 1.01)	0.96 (0.89, 1.03)	0.94 (0.87, 1.03)	0.95 (0.87, 1.05)
Multiple birth				
No	1.00	1.00	1.00	1.00
Yes	1.77 (1.41, 2.23)	1.58 (1.24, 2.03)***	1.98 (1.47, 2.66)	1.69 (1.22, 1.34) **
Breastfeeding initiation				
Within 1 h	1.00	1.00	1.00	1.00
After 1 h	4.57 (4.26, 4.90)	3.89 (3.61, 4.18)	4.58 (4.16, 5.03)	3.83 (3.48, 4.23) ***
Media exposure				
No	0.99 (0.93, 1.06)	1.15 (1.06, 2.24)**	1.18 (1.06, 1.30)	1.21 (1.07, 1.35) **
Yes	1.00	1.00	1.00	1.00
Parity				
Primiparous	1.00	1.00	1.00	1.00
Multiparous	0.80 (0.73, 0.87)	0.88 (0.80, 0.98)*	0.78 (0.70, 0.86)	0.89 (0.78, 1.01)
Grand multiparous	0.93 (0.85, 1.02)	0.90 (0.79, 1.03)	0.89 (0.80, 1.01)	0.91 (0.77, 1.08)
ANC visits				
No	1.00	1.00	1.00	1.00
One & two	0.60 (0.53, 0.69)	0.77 (0.67, 0.88)***	0.76 (0.62, 0.93)	0.89 (0.72, 1.11)
Three	0.43 (0.39, 0.49)	0.65 (0.56, 0.74)***	0.60 (0.49, 0.74)	0.81 (0.65, 1.01)
Four & above	0.44 (0.40, 0.49)	0.68 (0.60, 0.77)***	0.63 (0.52, 0.76)	0.85 (0.70, 1.41)
Place of delivery				
Home	1.00	1.00	1.00	1.00
Health facility	0.41 (0.38, 0.44)	0.43 (0.40, 0.47)***	0.46 (0.41, 0.51)	0.44 (0.39, 0.49) ***
Cesarean delivery				
No	1.00	1.00	1.00	1.00
Yes	2.09 (1.86, 2.33)	1.65 (1.45, 1.88)***	2.28 (1.96, 2.65)	1.63 (1.38, 1.93) ***
Size of the child at birth				
Small	1.40 (1.28, 1.54)	1.14 (1.03, 1.26)*	1.31 (1.15, 1.48)	1.15 (1.01, 1.32) *
Large	1.00	1.00	1.00	1.00
Average	0.93 (0.86, 1.01)	0.92 (0.83, 1.01)*	0.96 (0.87, 1.06)	0.95 (0.86, 1.06)
Distance from the health facility				
Big problem	1.00	1.00	1.00	1.00
Not a big problem	0.94 (0.88, 1.01)	1.02 (0.94, 1.09)	0.97 (0.88, 1.06)	0.98 (0.90, 1.08)
Residence				
Urban	1.00	1.00	0.84 (0.74, 0.95)	1.00
Rural	0.93 (0.86, 1.01)	0.84 (0.74, 0.90)***		0.78 (0.67, 0.91) **
Community-level women education				
Low	1.00	1.00	1.00	1.00
High	1.10 (1.01, 1.18)	1.10 (1.01, 1.21)	1.14 (1.02, 1.27)	1.10 (0.98, 1.24)
Community poverty level				
Low	1.00	1.00	1.00	1.00
High	0.89 (0.82, 0.97)	0.90 (0.82, 0.99)*	0.90 (0.81, 1.01)	0.95 (0.84, 1.06)
Community-level of ANC utilization				
High	1.00	1.00	1.00	1.00
Low	0.76 (0.67, 0.85)	0.84 (0.74, 0.95)*	0.73 (0.64, 0.84)	0.86 (0.71, 1.01)
Middle	1.08 (0.97, 1.20)	1.10 (0.99, 1.23)	1.01 (0.89, 1.15)	1.14 (1.01, 1.30)
Community-level media exposure				
Low	1.17 (1.07, 1.27)	1.11 (1.01, 1.23)*	1.30 (1.16, 1.45)	1.22 (1.09, 1.36) **
High	1.00	1.00	1.00	1.00

Note: *ANC* Antenatal Care, *AOR* Adjusted Odds Ratio, *CI* Confidence Interval, * = *p* value< 0.05, ** = *p* value≤0.01, *** = *p* value< 0.001

#### Random effect analysis and model comparison

Table 5 revealed the random effect analysis for the model with the weighted data. The ICC value in the null model indicates 9.3% of the total variations of prelacteal feeding practice were due to the difference between clusters. Besides, the high MOR value in the null model which was 1.74 revealed that when we randomly select mothers from two clusters, mothers from a high-risk cluster had 1.74 times more likely to practice prelacteal feeding as compared to mothers from a low-risk cluster. Moreover, the PCV in the final model revealed that about 13.4% of the variability in prelacteal feeding practice was explained both by individual and community-level factors. Regarding model fitness, model 3 was the best-fit model since it had the lowest deviance (Table 5).

**Table 5:** Community-level variability of prelacteal feeding practice and model comparison

Parameter	Null model	Model 1	Model 2	Model 3
Community-level variance [SE]	0.341 (0.036)	0.302 (0.031)	0.329 (0.034)	0.295 (0.031)
ICC	0.093	0.084	0.091	0.082
MOR	1.74 (1.65, 1.85)	1.69 (1.60, 1.78)	1.72 (1.64, 1.83)	1.67 (1.59, 1.77)
PCV (%)	Reference	11.4	3.5	13.5
Model fitness				
Deviance	23,918	21,868	23,840	21,792

Note; *ICC* Intraclass Correlation Coefficient, *MOR* Median Odds Ratio, *SE* Standard Error

## Discussion

This study aimed to assess the pooled prevalence and associated factors of prelacteal feeding practice in east Africa. The pooled prevalence of prelacteal feeding was 12%. The prevalence in this study is in line with a study done in Ethiopia [36], however, it is lower than reports from other studies [7, 8, 37–39] (Table 6). This discrepancy might be due to the difference in the study population, the variation in living conditions, and the difference in access to media and information across countries. This suggests strategies concerning suboptimal feeding patterns are decreased over time due to the expanded utilization of maternal health services.

**Table 6:** Previous study findings on prelacteal-feeding practice

Authors	Title of the article	Findings	
		Prevalence	Factors associated with prelacteal feeding
Nguyen, 2013 [7]	Prelacteal feeding practices in Vietnam: challenges and associated factors	73.5%	Cesarean section (AOR: 2.94; 95% CI: 2.39, 3.61)
Berde, 2013 [8]	Determinants of prelacteal feeding practices in urban and rural Nigeria	Urban Nigeria (49.8%) & Rural Nigeria (66.4%)	No education and primary educational status ((AOR: 1.48; 95% CI:1.07, 2.04 and AOR: 1.31; 95% CI: 1.02, 1.69, respectively) Home delivery (AOR: 1.53; 95% CI:1.24, 1.89) Cesarean Delivery (AOR: 1.87; 95% CI:1.25, 2.80) Multiple birth (AOR: 2.37; 95% CI:1.14, 4.95)
El-Gilany, 2014 [10]	Newborn first Feed and prelacteal feeds in Mansoura, Egypt	58%	Urban residence (AOR: 3.8; 95%CI: 2.4, 6.0), Maternal education (AOR: 1.5; 95%CI: 1.1, 2.3), Father’s education secondary (AOR: 3.0; 95%CI: 1.7, 5.3); receiving ANC visits at private clinics and no antenatal care; Caesarean section (AOR: 2.1; 95%CI: 1.2, 3.2); female babies (AOR: 1.7; 95%CI: 1.1, 3.2), and low birth weight (AOR: 4.2; 95%CI: 1.6, 11.2)
Khanal, 2016 [12]	Prevalence and factors associated with prelacteal feeding in Western Nepal	(30.6%)	Higher parity (AOR: 2.05; 95% CI: 1.18, 3.54), low birth weight (AOR: 1.97; 95% CI: 1.23, 3.16), a cesarean delivery (AOR: 3.70; 95% CI: 2.37, 5.80), and wealthy status (AOR: 2.49; 95% CI: 1.52, 4.06)
Berde, 2017 [15]	Risk factors for prelacteal feeding in sub-Saharan Africa: a multilevel analysis of population data from twenty-two countries	32·2%	Cesarean section (AOR: 2·25; 95% CI: 2·06, 2·46). Other factors are also significantly associated with an increased likelihood of prelacteal feeding such as the mother’s lower educational status, lower number of ANC visits, home delivery, multiple birth, male infant, and having a small-sized baby at birth. Besides, belonging to lower quintiles decrease the odds of prelacteal feeding.
Ogah, 2012 [20]	A cross-sectional study of prelacteal feeding practice among women attending Kampala International University Teaching Hospital Maternal And Child Health Clinic, Bushenyi, Western Uganda.	31.3%	Delay in initiating breastfeeding increases the prelacteal feeding practice
Agho, 2016 [26]	Trends and predictors of prelacteal feeding practices in Nigeria (2003–2013)	59.0%	Mothers with no schooling (AOR: 1.65; 95% CI: 1.33, 2.03), Younger mothers (aged 15–24 years), Mothers who delivered at home (AOR: 1.45; 95% CI: 1.23, 1.71), and Delivered by caesarean section (AOR: 1.91; 95% CI: 1.17, 3.13)
Belachew, 2016 [27]	Individual and community-level factors associated with introduction of prelacteal feeding in Ethiopia	28.92%	Caesarean delivery (AOR: 1.87; 95% CI: 1.28, 2.73), and late initiation of breastfeeding (AOR: 5.32; 95% CI: 4.65, 6.09). Higher economic status (AOR: 0.72; 95% CI: 0.54, 0.98), large birth size of child (AOR: 0.80; 95% CI: 0.68, 0.95), and high community ANC use (AOR: 58; 95% CI: 0.38, 0.87).
Gualu, 2016 [28]	Determinants of prelacteal feeding practice among postpartum mothers in Debre Markos town, Amhara regional state, Ethiopia, 2016	19.1%	Inability to read and write (AOR: 3.5; 95%CI: 1.14, 10.75), giving birth to a male (AOR: 2.8; 95% CI: 1.23, 6.37), home delivery (AOR: 4.4; 95% CI: 1.78, 10.85)
Tekaly, 2017 [36]	Prelacteal feeding practice and associated factors among mothers having children less than 2 years of age in Aksum town, central Tigray, Ethiopia	10.1% (95% CI: 7.3, 13%).	< 4 ANC visit (AOR: 10.55; 95% CI: 4.78, 23.40), Cesarean section (AOR: 4.38; 95% CI:1.72, 11.12)
Temesgen, 2018 [37]	Prelacteal feeding and associated factors in Ethiopia: systematic review and meta-analysis	25.29% (95% CI: 17.43, 33.15)	ANC visit (AOR: 0.25; 95% CI: 0.09, 0.69), Timely initiation of breastfeeding (AOR: 0.28; 95% CI: 0.21, 0.38) Urban residence (AOR: 0.47; 95% CI: 0.26, 0.86) Home birth (AOR: 3.93; 95% CI: 2.17, 7.10)
Amele, 2019 [38]	Prelacteal feeding practice and its associated factors among mothers of children age less than 24 months old in Southern Ethiopia	(20.6%) (95% CI: 17.5, 24.4)	Extended family type (AOR: 10.64; 95% CI: 1.05, 10.71) Lack of breastfeeding counseling (AOR: 5.16; 95% CI: 1.76, 15.13) and Mothers who avoid colostrum (AOR: 9.72; 95% CI: 3.46, 27.30)
Patel, 2013 [39]	Factors associated with prelacteal feeding and timely initiation of breastfeeding in hospital-delivered infants in India	16.9%	Lower maternal education (AOR: 2.13; 95% CI 1.06, 4.35), Muslim religion (AOR: 2.27; 95% CI: 1.18, 4.36), and Delivery by cesarean section (AOR: 2.56; 95% CI: 1.56, 4.19)
Wolde, 2019 [40]	Prelacteal feeding and associated factors among mothers having children less than 24 months of age, in Mettu district, Southwest Ethiopia	14.2% [95% CI: 12.0, 17.0]	No maternal education (AOR: 3.54; 95% CI: 1.7, 6.98), Single ANC visits (AOR: 6.87; 95% CI: 3.21, 14.73), Home delivery (AOR: 3.04; 95% Cl: 1.60, 5.75) and Cesarean delivery (AOR: 4.27; 95% CI: 2.28, 7.99)
Argaw, 2019 [41]	Factors associated with prelacteal feeding practices in Debre Berhan district, North Shoa, Central Ethiopia: a cross-sectional, community-based study	14.2% (95% CI: 11.00–17.00%)	Home delivery (AOR: 4.70; 95% CI: 2.56, 8.60) Delayed initiation of breastfeeding (AOR: 5.58; 95% CI: 3.21, 9.46). Mothers who can read and write (AOR: 0.46; 95% CI: 0.22, 0.98).
Asim, 2020 [42]	Prelacteal feeding practices in Pakistan: a mixed-methods study	64.7%	Birth at public health facilities (AOR: 0.46; 95% CI: 0.02, 0.95) Maternal primary education (AOR: 2.28; 95% CI: 1.35, 3.85), and Delayed breastfeeding initiation (AOR: 0.03; 95% CI: 0.01, 0.61).
Gebremeskel, 2020 [43]	Magnitude of prelacteal feeding and its associated factors among mothers having children less than one year of age: a community-based cross-sectional study in rural Eastern Zone, Tigray, Ethiopia	24.7%	Parity (AOR = 1.52; 95% CI: 1.04–2.23), late initiation of breastfeeding (AOR = 1.83; 95% CI: 1.30–2.59), and colostrum discard (AOR = 1.57, 95% CI: 1.06–2.33).
Gao, 2020 [44]	Trends in prelacteal feeding practices in rural Bangladesh from 2004 to 2019	88.0%	Being having maternal education, improvements in socioeconomic status, and exposure to media decreases the odds of prelacteal feeding.

Note: *ANC* Antenatal Care, *AOR* Adjusted Odds Ratio, *CI* Confidence Interval

This study also found the high heterogeneity, from 3% in Malawi to 39% in Comoros, of prelacteal feeding practice across east African countries. This may be due to the difference in the study period. For example, the data for Comoros was collected in 2012, while the data for Malawi was collected in 2015. Besides, the high heterogeneity in prelacteal feeding practice may be due to the sociocultural and socioeconomic differences among mothers in east African countries.

This study identified different factors that were associated with prelacteal feeding practice. In the unweighted data analysis, factors such as maternal education, multiple birth, media exposure, parity, ANC visit, place of delivery, delivery by cesarean section, birth size, residence, community-level of media exposure, community-level of ANC utilization, and community poverty level were associated with prelacteal feeding practice.

However, weighed data analysis identified multiple birth, the timing of breastfeeding initiation, media exposure, place of delivery, delivery by cesarean section, size of the child at birth, residence, and community level of media exposure as predictors of prelacteal feeding practice. This finding is consistent with different studies done elsewhere (Table 6). We prefer to discuss the results we get from the weighted data, which is necessary when we analyze DHS data [32]. Weighting preserves the representativeness of data and it helps to get standard and appropriate statistical estimate (robust standard error) [32]. Therefore, we give stress to the findings from weighed analysis and the interpretations and discussions, in this paper, are based on the weighted data.

Institutional delivery was associated with lower odds of prelacteal feeding practice. This is consistent with studies done in Ethiopia, Nigeria, and Pakistan [26, 40–42]. This might be justified as many health centers and hospitals ensure breastfeeding counseling during pregnancy, delivery, and postpartum periods to deter prelacteal feeding practices [45–48]. Another possible reason for introducing prelacteal feeding might be since mothers who delivered at home have no the opportunity to access health information about safe breastfeeding practices.

Besides, a woman who delivered by cesarean section was more likely to provide prelacteal feeding. This is in agreement with studies conducted elsewhere [7, 12, 36, 39, 40]. This might be because those mothers may be difficult to give breast milk since they are still recovering from pain, immobilization, and tiredness. This indicates that physicians may not be equipped with the appropriate skills to support mothers under such circumstances.

Mother with a multiple birth was associated with a higher likelihood of prelacteal feeding practice. This is in agreement with a study done in sub-Saharan Africa [15]. This might be because the mother with multiple births perceives their breast milk as insufficient and more likely to practice prelacteal feeding. Regarding the timing of breastfeeding initiation, mothers who had delayed initiation of breastfeeding had higher odds of prelacteal feeding practice compared with their counterparts. This is supported by studies done in Ethiopia, Uganda, and Pakistan [20, 28, 41–43]. This may be justified, as the time between birth and breastfeeding initiation increase, there would be more room for malpractices such as prelacteal feeding.

The study at hand revealed that mothers with a small-sized baby had a higher likelihood of prelacteal feeding practice compared to mothers with large-sized babies. This is in concordance with different studies done elsewhere [10, 12, 15, 27]. This may be due to the misconception that small-sized babies will benefit from other foods and liquids.

Mothers who had exposure to different media and mothers from communities with a higher level of media exposure had lower odds of prelacteal feeding practice as compared to their counterparts. This finding is in line with a study done in Nigeria [44]. This may be because disseminating information about the impacts of prelacteal feeding through different media could prevent prelacteal feeding practice. This suggests that printing and electronic mass media play a significant role in fostering optimal breastfeeding practices.

Moreover, women from rural areas had lower odds of prelacteal feeding practice compared to those from urban areas. This is in line with a study done in Egypt [10]. This might be due to the recent expansion of health extension programs among rural people, which increases women’s level of understanding about the impact of prelacteal feeding on child health.

### Strength and limitations of the study

This study was based on the pooled analysis of the East Africa countries DHS. It was based on a multilevel analytical approach that can able to identify both individual and community-level factors that were associated with prelacteal feeding practice. Also, appropriate estimation adjustments such as weighting were applied. Therefore, the findings of this study will provide important insights to policymakers and governmental and non-governmental organizations to design the most appropriate interventions at both individual and community levels.

However, this study was not without limitations, in which while interpreting the study findings should be with caution. First, the outcome variable, prelacteal feeding practice was assessed based on the maternal self-report and therefore there might be a recall bias. Second, DHS did not collect some information such as maternal beliefs, misconceptions, and knowledge towards breastfeeding that were evidenced to influence prelacteal feeding practice. Third, the influence of medicines, including those used for cesarean sections are not assessed. Finally, since it was a cross-sectional study we are unable to assure the temporal relationship between prelacteal feeding practice and important independent variables such as the timing of initiation of breastfeeding.

## Conclusion

In this study, the pooled prevalence of prelacteal feeding is high and still needs strengthening of interventions on appropriate breastfeeding practices. Both individual and community level variables were associated with prelacteal feeding practice. Of individual-level factors, home delivery, multiple birth, cesarean delivery, non-exposure to media, delayed initiation of breastfeeding, and being small-sized baby were associated with higher odds of prelacteal feeding practice. Among community-level factors, rural residence, and higher community-level of media exposure were associated with lower odds of prelacteal feeding practice. Therefore, individual and community-level interventions that encourage mothers to deliver in the health facility and promote timely initiation of breastfeeding are needed to reduce prelacteal feeding practices in east Africa. Moreover, media campaigns regarding this harmful traditional practice are recommended.

## Data Availability

We included all result-based data within the manuscript and the data set can be accessed online from www.measuredhs.com/data.

## Abbreviations

ANC: Antenatal Care
AOR: Adjusted Odds Ratio
ICC: Intraclass Correlation coefficient
MOR: Median Odds Ratio
PCV: Proportional change in Variance
VIF: Variance Inflation Factor

## References

[1] World Health Organization (2009). Infant and young child feeding: model chapter for textbooks for medical students and allied health professionals.

[2] Tessema M, Belachew T, Ersino G (2013). Feeding patterns and stunting during early childhood in rural communities of Sidama, South Ethiopia. Pan Afr Med J.

[3] UNICEF (2016). From the first hour of life: making the case for improved infant and young child feeding everywhere.

[4] Pem D (2015). Factors affecting early childhood growth and development: golden 1000 days. J Adv Pract Nurs.

[5] World Health Organization (2009). Global health risks: mortality and burden of disease attributable to selected major risks.

[6] Lakati A, Makokha O, Binns C, Kombe Y (2011). The effect of pre-lacteal feeding on full breastfeeding in Nairobi, Kenya. East Afr J Public Health.

[7] Nguyen PH, Keithly SC, Nguyen NT, Nguyen TT, Tran LM, Hajeebhoy N (2013). Prelacteal feeding practices in Vietnam: challenges and associated factors. BMC Public Health.

[8] Berde AS, Yalcin SS, Ozcebe H, Uner S, Caman OK (2017). Determinants of pre-lacteal feeding practices in urban and rural Nigeria; a population-based cross-sectional study using the 2013 Nigeria demographic and health survey data. Afr Health Sci.

[9] Dalal S, Bansal M, Pande K (2016). Study of infants deaths and pre lacteal feeding practices using verbal autopsy as a tool in deharadun. Natl J Community Med.

[10] El-Gilany A-H, Abdel-Hady DM (2014). Newborn first feed and prelacteal feeds in Mansoura, Egypt. BioMed Res Int.

[11] Bansal S, Lalit U, Mahajan R (2016). Prevalence of prelacteal feeding among newborn in rural area. Int J Curr Med Appl Sci.

[12] Khanal V, Lee AH, Karkee R, Binns CW (2016). Prevalence and factors associated with prelacteal feeding in Western Nepal. Women Birth.

[13] Legesse M, Demena M, Mesfin F, Haile D (2014). Prelacteal feeding practices and associated factors among mothers of children aged less than 24 months in Raya Kobo district, North Eastern Ethiopia: a cross-sectional study. Int Breastfeed J.

[14] Boccolini CS, Pérez-Escamilla R, Giugliani ERJ, Boccolini P (2015). Inequities in milk-based prelacteal feedings in Latin America and the Caribbean: the role of cesarean section delivery. J Hum Lact.

[15] Berde AS, Ozcebe H (2017). Risk factors for prelacteal feeding in sub-Saharan Africa: a multilevel analysis of population data from twenty-two countries. Public Health Nutr.

[16] Karkee R, Lee AH, Khanal V, Binns CW (2014). Initiation of breastfeeding and factors associated with prelacteal feeds in Central Nepal. J Hum Lact.

[17] Teshome B, Kogi-Makau W, Getahun Z, Taye G. Magnitude and determinants of stunting in children underfive years of age in food surplus region of Ethiopia: the case of west gojam zone. Ethiop J Health Dev. 2009;23(2). 10.4314/ejhd.v23i2.53223.

[18] Bekele Y, Mengistie B, Mesfine F (2014). Prelacteal feeding practice and associated factors among mothers attending immunization clinic in Harari region public health facilities, eastern Ethiopia. Open J Prev Med.

[19] Hurley WL, Theil PK (2011). Perspectives on immunoglobulins in colostrum and milk. Nutrients.

[20] Ogah A, Ajayi A, Akib S, Okolo S (2012). A cross-sectional study of pre-lacteal feeding practice among women attending Kampala International University teaching hospital maternal and child health clinic, Bushenyi, Western Uganda. Asian J Med Sci.

[21] Ludvigsson JF (2003). Breastfeeding intentions, patterns, and determinants in infants visiting hospitals in La Paz, Bolivia. BMC Pediatr.

[22] Khanal V, Adhikari M, Sauer K, Zhao Y (2013). Factors associated with the introduction of prelacteal feeds in Nepal: findings from the Nepal demographic and health survey 2011. Int Breastfeed J.

[23] Raheem RA, Binns CW, Chih HJ, Sauer K (2014). Determinants of the introduction of prelacteal feeds in the Maldives. Breastfeed Med.

[24] Ibadin O, Ofili N, Monday P, Nwajei C (2013). Prelacteal feeding practices among lactating mothers in Benin City, Nigeria. Nigerian J Paediatr.

[25] Engebretsen IMS, Nankabirwa V, Doherty T, Diallo AH, Nankunda J, Fadnes LT, Ekström E-C, Ramokolo V, Meda N, Sommerfelt H (2014). Early infant feeding practices in three African countries: the PROMISE-EBF trial promoting exclusive breastfeeding by peer counsellors. Int Breastfeed J.

[26] Agho KE, Ogeleka P, Ogbo FA, Ezeh OK, Eastwood J, Page A (2016). Trends and predictors of prelacteal feeding practices in Nigeria (2003–2013). Nutrients.

[27] Belachew AB, Kahsay AB, Abebe YG (2016). Individual and community-level factors associated with introduction of prelacteal feeding in Ethiopia. Arch Public Health.

[28] Gualu T, Dilie A, Haile D, Abate A (2017). Determinants of prelacteal feeding practice among postpartum mothers in Debre Markos town, Amhara regional state, Ethiopia, 2016. Nutr Diet Suppl.

[29] Croft TN, Marshall AMJ, Allen CK (2018). Guide to DHS Statistics.

[30] The DHS program: DHS Methodology. Available from: http://dhsprogram.com/What-We-Do/Survey-Types/DHS-Methodology.cfm.

[31] Central statistical agency (CSA)[Ethiopia] and ICF (2016). Ethiopia demographic and health survey, Addis Ababa, Ethiopia and Calverton, Maryland, USA.

[32] The DHS Program: Sampling and Weighting with DHS Data. 2015. Avialable at https://blog.dhsprogram.com/sampling-weighting-at-dhs/

[33] Austin PC, Merlo J (2017). Intermediate and advanced topics in multilevel logistic regression analysis. Stat Med.

[34] Sommet N, Morselli D (2017). Keep calm and learn multilevel logistic modeling: a simplified three-step procedure using stata, R, Mplus, and SPSS. Int Rev Soc Psychol.

[35] Merlo J, Chaix B, Ohlsson H, Beckman A, Johnell K, Hjerpe P, Råstam L, Larsen K (2006). A brief conceptual tutorial of multilevel analysis in social epidemiology: using measures of clustering in multilevel logistic regression to investigate contextual phenomena. J Epidemiol Community Health.

[36] Tekaly G, Kassa M, Belete T, Tasew H, Mariye T, Teshale T (2018). Pre-lacteal feeding practice and associated factors among mothers having children less than two years of age in Aksum town, Tigray, Ethiopia, 2017: a cross-sectional study. BMC Pediatr.

[37] Temesgen H, Negesse A, Woyraw W, Getaneh T, Yigizaw M (2018). Prelacteal feeding and associated factors in Ethiopia: systematic review and meta-analysis. Int Breastfeed J.

[38] Amele EA, Birhanu WD, Desta KW, Woldemariam EB (2019). Prelacteal feeding practice and its associated factors among mothers of children age less than 24 months old in Southern Ethiopia. Ital J Pediatr.

[39] Patel A, Banerjee A, Kaletwad A (2013). Factors associated with prelacteal feeding and timely initiation of breastfeeding in hospital-delivered infants in India. J Hum Lact.

[40] Wolde TF, Ayele AD, Takele WW (2019). Prelacteal feeding and associated factors among mothers having children less than 24 months of age, in Mettu district, Southwest Ethiopia: a community based cross-sectional study. BMC Res Notes.

[41] Argaw MD, Asfaw MM, Ayalew MB, Desta BF, Mavundla TR, Gidebo KD, Frew AH, Mitiku AD, Desale AY (2019). Factors associated with prelacteal feeding practices in Debre Berhan district, north Shoa, Central Ethiopia: a cross-sectional, community-based study. BMC Nutr.

[42] Asim M, Ahmed ZH, Hayward MD, Widen EM (2020). Prelacteal feeding practices in Pakistan: a mixed-methods study. Int Breastfeed J.

[43] Gebremeskel SG, Gebru TT, Kassahun SS, Gebrehiwot BG (2020). Magnitude of prelacteal feeding and its associated factors among mothers having children less than one year of age: a community-based cross-sectional study in rural eastern zone, Tigray, Ethiopia. Adv Public Health.

[44] Gao Y, Palmer A, Thorne-Lyman A, Shaikh S, Ali H, Tong H, et al. Trends in prelacteal feeding practices in rural Bangladesh from 2004–2019. Curr Dev Nutr. 2020;4(2). 10.1093/cdn/nzaa053_034.

[45] Lind JN, Ahluwalia IB, Perrine CG, Li R, Harrison L, Grummer-Strawn LM (2014). Prenatal breastfeeding counseling--pregnancy risk assessment monitoring system, United States, 2010. MMWR Suppl.

[46] World Health Organization: Counselling for maternal and newborn health care: A handbook for building skills. World Health Organization; 2010. 26158182

[47] Edwards RA, Colchamiro R, Tolan E, Browne S, Foley M, Jenkins L, Mainello K, Vallu R, Hanley LE, Boisvert ME, Forgit J (2015). Online continuing education for expanding clinicians’ roles in breastfeeding support. J Hum Lact.

[48] World Health Organization (2018). Guideline: counselling of women to improve breastfeeding practices.

